# Nitrogen-Based Heterocyclic Compounds: A Promising Class of Antiviral Agents against Chikungunya Virus

**DOI:** 10.3390/life11010016

**Published:** 2020-12-30

**Authors:** Andreza C. Santana, Ronaldo C. Silva Filho, José C. J. M. D. S. Menezes, Diego Allonso, Vinícius R. Campos

**Affiliations:** 1 Departamento de Química Orgânica, Campus do Valonguinho, Instituto de Química, Universidade Federal Fluminense, Niterói, Rio de Janeiro 24020-141, Brazil; andrezasantana@id.uff.br (A.C.S.); ronaldocouto@id.uff.br (R.C.S.F.); 2 Section of Functional Morphology, Faculty of Pharmaceutical Sciences, Nagasaki International University, 2825-7 Huis Ten Bosch, Sasebo, Nagasaki 859-3298, Japan; 3 Research & Development, Esteem Industries Pvt. Ltd., Bicholim, Goa 403 529, India; 4 Departamento de Biotecnologia Farmacêutica, Faculdade de Farmácia, Universidade Federal do Rio de Janeiro, Rio de Janeiro 21941-902, Brazil

**Keywords:** nitrogen heterocyclic, Chikungunya virus, antiviral, arboviruses

## Abstract

Arboviruses, in general, are a global threat due to their morbidity and mortality, which results in an important social and economic impact. Chikungunya virus (CHIKV), one of the most relevant arbovirus currently known, is a re-emergent virus that causes a disease named chikungunya fever, characterized by a severe arthralgia (joint pains) that can persist for several months or years in some individuals. Until now, no vaccine or specific antiviral drug is commercially available. Nitrogen heterocyclic scaffolds are found in medications, such as aristeromycin, favipiravir, fluorouracil, 6-azauridine, thioguanine, pyrimethamine, among others. New families of natural and synthetic nitrogen analogous compounds are reported to have significant anti-CHIKV effects. In the present work, we focus on these nitrogen-based heterocyclic compounds as an important class with CHIKV antiviral activity. We summarize the present understanding on this class of compounds against CHIKV and also present their possible mechanism of action.

## 1. Introduction

Mosquito-borne viruses represent a serious health problem worldwide due to a number of factors, including the vectors’ growing geographic expansion and the easy mobility of humans across all continents, which culminates in a perfect scenario for virus propagation [1]. Among the arboviruses that cause human disease, Chikungunya virus (CHIKV) is one of the most relevant, mainly because of its great social and economic impact resultant from its high morbidity, although it is rarely associated with death. It was first isolated in the 1950s in the region currently known as Tanzania, and since then has been responsible for some sporadic outbreaks in the African and Asian continents [2]. CHIKV is transmitted by mosquitos of the genus *Aedes* spp., most commonly by *Aedes aegypti* and *Aedes albopictus* [3].

The adaptation of CHIKV to replicate in *Aedes albopictus* has expanded its spread around the world, resulting in outbreaks not only in Africa and Southeast Asia, but in other continents such as Europe (mainly in Italy and France) and in the Americas [4]. In the Americas, the first CHIKV outbreak was reported in 2013 on the Caribbean island of Saint Martin, which resulted in more than 1.2 million cases [5]. Since then, other countries such as Brazil, have documented persistent outbreaks of CHIKV. In the second half of 2020 alone, approximately 81,000 cases of chikungunya fever have been reported across the Americas, and 97% of the cases were in Brazil [6,7].

CHIKV is a member of the *Togaviridae* family and of the *Alphavirus* genus, and is contained in the Semliki forest antigenic complex, which has other alphaviruses, such as: Ross River, O’nyong-nyong, Getah, Bebaru, Semliki forest, and Mayaro viruses, which are endemic in South America [7]. Clinical symptoms are observed in almost all infected individuals and more than half of the patients have persistent polyarthritis, which might last several months or years after the acute viral infection [8].

Despite causing morbidity, there is no therapeutic treatment available on the market against CHIKV. Some compounds with a broad antiviral activity such as ribavirin, harringtonine, and interferon-alfa (IFN-α) showed great efficiency against CHIKV only in vitro [9]. On the other hand, the use of chloroquine exhibited a dose-dependent and time-dependent inhibitory effect on CHIKV replication in vitro, but clinical studies have failed to prove its effectiveness in infected patients [10,11].

In clinical practice, treatment is strictly limited to symptom relief, with the use of antipyretics, analgesics, corticosteroids, and non-steroidal anti-inflammatory drugs. In some patients with severe arthralgia and polyarthritis, the administration of methotrexate and sulfasalazine which are called disease modifying anti-rheumatic drugs, is necessary [12].

Therefore, it becomes clear that there is an urgency and a necessity to discover new classes of compounds that can be used in clinics to treat CHIKV infection. In the search for new candidates with anti-CHIKV activity, nitrogen-based heterocyclic derivatives, such as pyrimidine, pyrazine, and purine, have emerged as promising compounds. Scientific literature points to a very interesting potential of these scaffolds against CHIKV infection therapy. Therefore, this work aims to review the existing literature for natural and synthetic nitrogen-based heterocyclic derivatives and their potential CHIKV antiviral activity.

## 2. CHIKV Infection

CHIKV has a single-strand, positive-polarity RNA genome, whose size varies between 9.7 and 12 kb [13]. The viral particles, which are often spherical, have a diameter of 70 nm and an isometric nucleocapsid of 40 nm [14]. The proteins are encoded by two open reading frames (ORFs) in which the first two-thirds of the genome from the 5′ end is responsible for expression of the non-structural proteins (NSPs) named nsP1, nsP2, nsP3, and nsP4; whereas the last one-third located at the 3′ end codifies a subgenomic RNA that originates from three structural proteins and two peptides: capsid (C), enveloped glycoprotein 1 (E1), glycoprotein enveloped 2 (E2), E3, and 6K [1,15,16].

Glycoproteins E1 and E2, which form heterodimers, are found on the viral surface and are involved in the target cell recognition [10,12,17]. After interaction with a specific receptor in the cell membrane, the virus particle undergoes endocytosis in vesicles covered by clathrins. The pH acidification in the endosome causes a tridimensional rearrangement of the E1 subunit culminating in the exhibition of the fusion peptide, which will be responsible for the fusion of the envelope with the endosomal membranes and the consequent release of the nucleocapsid to the cytoplasm. Thereafter, the RNA genome gets exposed, and translation of NSPs begins. These NSPs are responsible for the replication complex assembly and perform all the steps necessary for successful virus replication. It is important to highlight that the NSPs are translated as a polyprotein that are further processed as nsP1, nsP2, nsP3, and nsP4 by the action of the viral protease nsP2 [10,13].

The nsP1 manages the enzymatic activities of guanine-7-methyltransferase and guanylyl transferase. It is also associated with membranes and probably acts as a scaffold to attach the viral replication complex (non-structural proteins) to the host membranes. The capping of viral genomes is critical because it has its importance in translating proteins from genomic and subgenomic RNAs, making it a potential target for antiviral drugs because it is specific and less prone to side effects [11,18,19].

NsP2 has the following enzymatic functions: RNA helicase, nucleoside triphosphatase (NTPase), and RNA-dependent RNA-triphosphatase-5′RNA activity, despite its protease activity. This protein is essential for viral viability [16,20]. The nsP3 macrodomain acts as part of the replicase unit and is present in RNA synthesis. It is subdivided into three domains: N-terminal that connects with negatively charged polymers, the one unique to alphavirus (AUD) and hypervariable C-terminal (HVR), which are responsible for the metabolism of the ADP ribose derivatives, which regulate functions in the cell. NsP4 acts as an RNA-dependent RNA polymerase that synthesizes new viral RNAs from the pre-existing RNA genome [3,12,18,21].

The lack of proofreading activity of the nsP4, which is responsible for high mutation rates [22], in association with the A226V substitution at E1 protein, may be related to a greater virus virulence and allow its best adaptation to replicate in *Aedes albopictus* mosquitoes, which explains the rapid spread of the disease between 2004 and 2010 to many countries around the Indian Ocean [23]. To date, four CHIKV genotypes have been described: Asian, East Indian, West African, and East/Central/South African. The Asian genotype was first detected in the Caribbean region in late 2013 and then spread throughout Central America [14].

## 3. Overview of CHIKV Drugs and the Versatility of Nitrogen-Based Heterocycles Derivatives

The ideal treatment of CHIKV-infected patients needs a safe, specific, and efficient antiviral drug that could both eliminate viral replication and block systemic and joint inflammation due to viral infection. Drugs commercially available for other diseases including fever-like illnesses are often used to manage CHIKV symptoms, such as orlistat, chloroquine, favipiravir, arbidol, and ribavirin. However, due to lack of specificity of these drugs against CHIKV infection, they fail to promote complete viral clearance and abrogate its clinical manifestation [8].

The development of a wide spectrum antiviral drugs against different arboviruses, including CHIKV, was for a long time encouraged by the fact they share several mechanisms of pathogenicity. In practice, this strategy has proven quite challenging, since each virus has different cell tropism, which elicits distinct inflammatory responses, as well as promotes different mechanisms of immune evasion. The structural and functional diversity of viral enzymes often used as pharmacological targets, such as the RNA-dependent RNA polymerase (nsP4) and protease (nsP2), significantly alters the affinity and activity of polymerase or protease inhibitors, respectively, which, in the end, drives the antiviral development to a virus-specific pathway [10,13,24,25,26,27,28,29,30].

**Figure 1:**
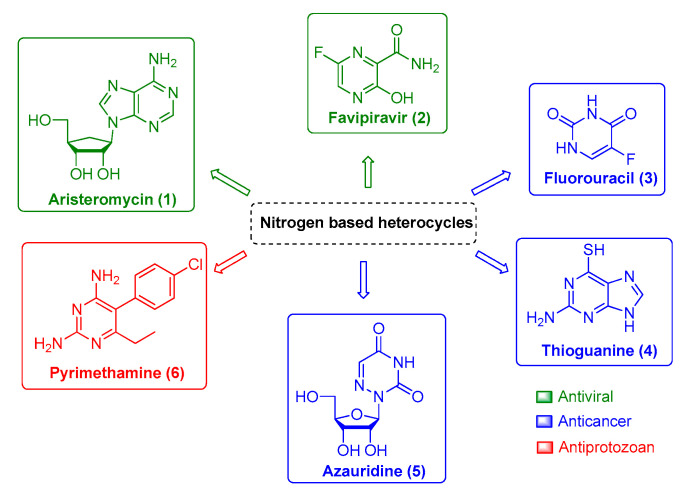
Nitrogen-based heterocycles derivatives with different biological activities.

Therefore, finding potent and efficient antivirals with novel scaffolds that have the potential to specifically interact and impair viral enzymatic activities of a single viral family or viral genus is the pursued path toward successful drug development. In this context, the nitrogen-based heterocyclic analogs are an important class of organic compounds that have a privileged position. They are a valuable source for therapeutic agents, which is corroborated by the increasing attention received by these compounds over the last decades. Among them, pyrimidine and its derivatives have received great attention due to their biological and pharmacological activities against viral infection, such as aristeromycin (**1**) and favipiravir (**2**); as well as anticancer drug, such as fluorouracil (**3**), thioguanine (**4**), and azauridine (**5**); and as antiprotozoal drugs, such as pyrimethamine (**6**) (Figure 1). Moreover, this class of molecules is usually reported as being druggable and safe for human use, which makes it more attractive for further development. Therefore, in the present review, we will summarize natural and synthetic nitrogen-based heterocyclics and their derivatives with reported anti-CHIKV activity [9,11,19,25,31,32,33,34,35,36,37,38,39] and explore the potential of these molecules as an interesting class for antiviral drug development.

### 3.1. Nitrogen Heterocycle–Coumarin Hybrid Compounds

Several natural compounds based on herbs and plant extracts were investigated for activity against CHIKV [40,41]. Among natural products, coumarin has gained much attention due its pharmacological properties against several diseases. Coumarins with biological activities can be found in various parts of the plant, as seeds, leaves, and roots and also in distinct plant families, such as *Apiaceae*, *Rutaceae*, *Clusiaceae*, and *Umbelliferae* [42]. Natural coumarin derivatives have been described as having anticoagulant [43], antibacterial, antioxidant, and anticancer properties [44].

Coumarin has been known to play an incremental role in targeting various cell pathways, inhibiting growth and replication, among other targets for a wide range of viruses. The anti-influenza activity of coumarin derivatives was associated with neuraminidase’s inhibition of the viral envelope protein [45]. In the case of the human immunodeficiency virus (HIV), coumarin analogs, isomesuol and mesuol (extracted from *Marila pluricostata*), could act via the phosphorylation of p65 (NF-kB) [46]. While in the hepatitis C virus (HCV), coumarins could act against its replication, increasing the interferon-mediated antiviral responses [47]. The activity of coumarins against dengue virus (DENV) has already been reported, but its mechanisms of action requires further investigation; however, initial studies point to the NS2B/NS3 complex as a possible coumarin target [48]. The activity of coumarin against CHIKV is still under investigation [42,49,50,51,52,53].

**Figure 2:**
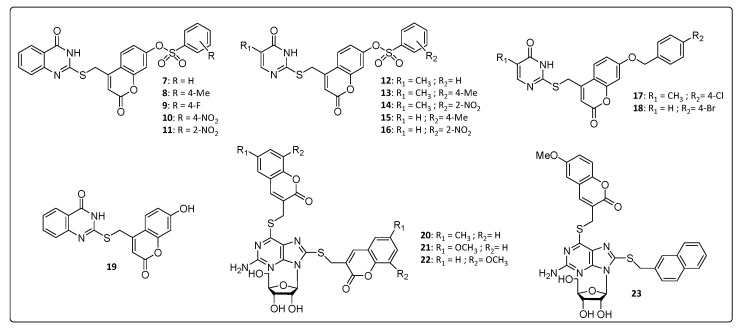
Nitrogen heterocycle–coumarin hybrid with potential activity against Chikungunya virus (CHIKV).

In Figure 2, we have summarized some coumarin–nitrogen-based heterocyclic derivatives that were reported to have anti-CHIKV activity. Compounds **7**, **8**, **11**, **14** and **16** were the most potent inhibitors, with EC_50_ values ranging from 10.2 to 19.1 μM (concentration necessary to reduce 50% of viral infection in Vero cells), and with a selectivity index (SI) around 5.6 and 11.5 (Table 1). The calculation of the molecular lipophilicity quantified by logP is a major determinant for the absorption of the compound, distribution in the body, penetration through biological barriers, metabolism and excretion (ADME). An ideal logP range is generally between 0.4 and 5.0. Higher values are indicative of malabsorption or permeation of the components. For these pyrimidine–coumarin hybrids, the logP values remained in the range from 2.74 to 3.57 [34,36].

With respect to the benzenesulfonyl ring on coumarin, the *para*-methyl group at **8**, increased the potency and SI value when compared to that of the unsubstituted derivatives **7**. The same correlation can be done with nitro compound **11** that increased activity by 2 times, approximately, compared to the 4-position for the same substituent in **10**. While for pyrimidine analogs, **12**–**16**, the nitro group at 2-position on the benzenesulfonyl ring on coumarin showed the best activity. Overall, the quinazoline derivatives, **7**–**11**, were more active than the pyrimidine analogs, **12**–**16**, whose logP values are directly proportional to the activity presented **8** > **13** > **15** and **11** > **14** > **16** (Table 1). The presence of hydrophobic substituents at the 5 and 6 position of pyrimidine nucleus (**7**–**11**) was an important synthetic strategy, conferring greater activities against CHIKV and other viruses, such as HIV and HCV [34].

A series of thioguanine-coumarins derivatives were studied, and the methoxylated coumarins [54] (**21**, **22** and **23**) exhibited the best activities against CHIKV compared to the benzouracil coumarins (**9**–**19**). EC_50_ values were below 13.9 μM with a significant selectivity window covering SI values between 9.37 and 21.7, whereas the molecular lipophilicity of **20**, **21**, **22** and **23** were between 2.97 and 3.77. The replacement of the coumarinyl (**21**) by naphthyl group (**23**) resulted in no significant influence in the EC_50_ value, probably because of the similar size and planarity of the two substituents. In contrast, substitution of the naphthyl to benzyl (not shown) considerably decreased the EC_50_ values [54], confirming the importance of the size and planarity of the substituents for proper antiviral activity [54].

### 3.2. Aristeromycin Analogs

The purine analog, aristeromycin, is a naturally occurring molecule found in filamentous bacteria of the species *Streptomyces citricolor*. It provided potent activity against CHIKV, but its high cytotoxicity limited its therapeutic use [35,36]. Mechanistically, aristeromycin has been described as an inhibitor of the hydrolase S-adenosyl-L-homocysteine (SAH), which can also indirectly inhibit the activity of methyl transferases (MTase), which are responsible for capping the viral mRNA. Similar activity in CHIKV is mediated by the nsP1 protein [55].

**Figure 3:**
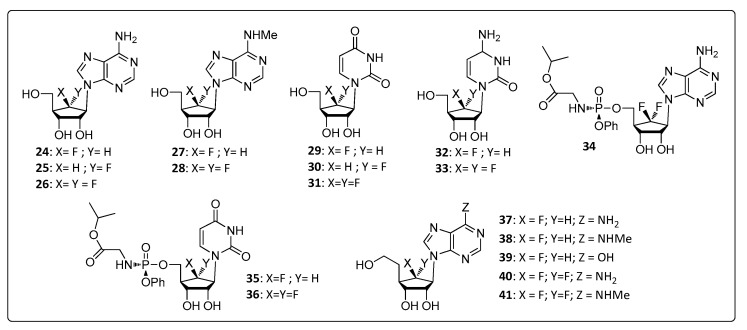
Aristeromycin analogs designed for antiviral activity against CHIKV.

Therefore, D-6′-fluorinated-aristeromycin analogs (Figure 3) were designed to assess the inhibition of the human SAH and correlate the potential antiviral activity against CHIKV by a probable inhibition of nsP1. It was found that the 6-amino-purines (**24**, **25**, **26**) showed interesting results for SAH inhibition or for anti-CHIKV activity when compared with aristeromycin and also with other purines (**27** and **28)**, pyrimidines (**29**–**33**), and phosphoramidates (**34**, **35**, **36**) as summarized in Table 2.

These data confirmed the antiviral activity against CHIKV and also suggested that the observed activity may be due to an indirect effect on the activity of nsP1. Compound **26** was one the most potent inhibitors for SAH (Table 2). Although compound **26** was less toxic than aristeromycin, substantial cytotoxicity, indicated that further adjustments were necessary prior to in vivo and human tests [36].

Among phosphoramidates, which were selectively designed to inhibit virus replication through the binding on the viral polymerase, compound **34** showed the best efficacy and broad-spectrum activity against CHIKV, Middle East respiratory syndrome–coronavirus (MERS-CoV), severe acute respiratory syndrome–coronavirus (SARS-CoV), and Zika virus (ZIKV) but failed in terms of toxicity [36].

The cytotoxicity of the compounds **26** and **34** is explained by their ability to be phosphorylated at 5′, acting as a nucleoside analog that interacts with both viral and host RNA polymerases blocking their activity or preventing host RNA elongation. For this reason, homoaristeromycin analogs with 5′ hydroxyl displacement susceptible to the phosphate transfer zone in kinases were developed. This family of compounds has the advantage of preventing 5′-phosphorylation by cell kinases, maintaining inhibitory activity in relation to SAH. Since the cytotoxicity of compound **1** can be attributed to 5′-phosphorylation, which occurs in the biological environment. Among the compounds of this family, derivative **37** stands out due to its high inhibition of SAH (IC_50_ = 0.36 μM), as well as its good inhibitory activity against CHIKV (EC_50_ = 0.12 μM) and low cytotoxicity (CC_50_ > 250 μM) [35].

Compound **38**, unlike **37**, showed similar levels of SAH inhibition (IC_50_ = 0.37 μM) but failed to block viral infection. This result sheds light on the direct role of aristeromycin and its analog on viral nsP1. Kovacikova and collaborators [23] figured out that combined mutations G230R and K299E in the CHIKV nsP1 conferred resistance to 6′-fluoro-homoneplanocin A (FHNA) and 6′-β-fluoro-homoaristeromycin (FHA). Moreover, in vitro enzymatic analyses with purified and active nsP1 from the alphavirus Semiliki Forest virus indicated that the oxidized (3′-keto) form of FHNA, obtained under nonreducing conditions and in the presence of NAD^+^, can directly inhibit nsP1 MTase activity. Together, these results confirmed that aristeromycin analogs might act directly on the nsP1 MTase activity, rather than just through an indirect effect on the host SAH hydrolase. In addition, the replacement of NH_2_ (**37**) by NHMe (**38**) significantly decreased its antiviral activity, suggesting that this region of the molecule was very important for the nsP1 inhibitory activity [35].

### 3.3. Ribonucleoside Derivatives

Nucleoside analogs as antiviral compounds are justified by the high probability of their absorption by human cells and incorporation into nucleic acids. This can block nucleic acid synthesis by preventing the incorporation of new nucleosides into the growing RNA strand inhibiting crucial processes of cell metabolism, such as cell division and, in the case of virus infection, the viral replication process [37].

**Figure 4:**
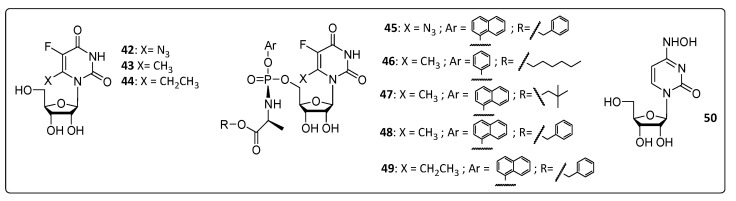
Structure of the ribonucleoside derivatives **42**–**50**.

Two series of ribonucleosides (**42**–**44** and **45**–**49**) were planned by Slusarczyk and co-workers (Figure 4), and the latter was based on the ProTide technology developed by McGuigan to improve the activity and to overcome resistance through the introduction of an amino acid ester and two lipophilic and biolabile groups linked to the phosphate group of the molecule, optimizing their membrane permeability and thus avoiding the need for nucleoside transporters [37]. Pyrimidine and its analogs may be involved in the inhibition of the host’s intracellular enzyme orotidine-5′-monophosphate decarboxylase rather than acting against specific viral enzymes, and this may explain why most of the compounds tested in the cytopathic-based assay in Vero cells did not exhibit antiviral activity against CHIKV (EC_50_ > 200 µM; Table 3) [37].

On the other hand, β-D-N^4^-hydroxycytidine (**50**) exhibited good antiviral activity against CHIKV at a lower concentration than favipiravir (**2**) (EC_50_ = 0.2 µM), which was used as the gold standard for both the Asian and African CHIKV strains. It was suggested that its performance occurs via the inhibition of the initial negative-stranded RNA synthesis or through chain termination or mutagenesis, which can, in turn, interfere with the correct formation of the replicase complex. The fact is that CHIKV replication was remarkably inhibited by this compound. The calculated CC_50_ of compound **50** was 7.7 µM in Vero cells, which is at least 38 times higher than its EC_50_. The cytotoxicity of **50** changes drastically according to cell line used in the assay, for peripheral blood mononuclear cells (PBMCs) it was 30.6 µM and for lymphoblastic cells originally derived from a child with acute lymphoblastic leukemia (CEM), it was 2.5 µM. In all cases, the CC_50_ was at least 10 times higher than the EC_50_ (Tabel 3) [38].

### 3.4. Piperazinyl–Pyrimidine Derivatives

**Figure 5:**
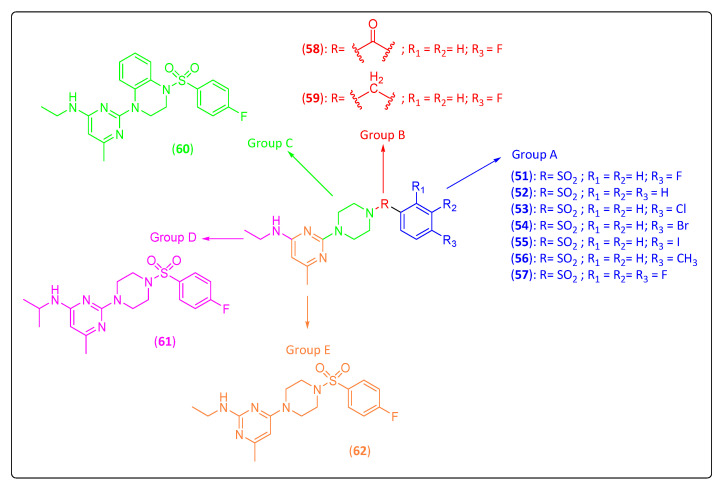
Piperazinyl–pyrimidine analogs (**51**–**62**) as inhibitors of Chikungunya virus.

The piperazinyl–pyrimidine derivatives (**52**–**62**) were planned by optimization of the compound **51**, which was identified by high-throughput screening by the Center for Innovation and Stimulation of Drug Discovery and by the University of Leuven [9]. To optimize compound **51**, systematic structural variations (shown in Figure 5) were performed in its structure, which was divided into five parts to achieve an increase in antiviral activity and a decrease in cytotoxicity. For group A (modifications on, or replacement of, the 4-fluorophenyl ring), compounds **53**, **54** and **55** can be highlighted since all these compounds were more active than the standard **51** (Table 4). Chlorine-substituted **53** gave the best result with an EC_50_ of 5 µM, and iodine-substituted **55** (EC_50_ of 5.9 µM) and bromine-substituted **54** (EC_50_ = 7.1 µM) were less active than **53**. In order to assess the influence of the sulfonamide linker, group B was designed to replace it with an amide (**58**) or a methyl group (**59**). Both compounds were more than twice as potent as **51**, derivative **58** had an EC_50_ of 4 µM, and **59** had an EC_50_ of 2.5 μM. Substituents of the piperazine linker (Group C) were introduced to investigate the function of this group, and the results showed that the prepared analog (**60**) was less active (EC_50_ of 16 µM) than **51**. Therefore, according to the authors, 1,4-piperazine was the better choice to reach a potent antiviral activity [9]. For group D, the replacement of the ethylamine side chain with isopropylamine provided derivative **61**, which demonstrated good activity (EC_50_ of 9.5 µM) against CHIKV compared to compound **51**. However, this result was also accompanied by increased cytotoxicity (CC_50_ = 66.4 μM), indicating that the ethylamine side chain is essential for antiviral activity. Finally, in group E, we investigated whether the methyl group as a substituent on the pyrimidine ring is essential or not for the antiviral activity. The results showed that derivative **62** also demonstrated good activity (EC_50_ of 3.2 µM) against CHIKV compared to **51**. Nevertheless, the cytotoxicity of this compound was increased (Table 4) [9].

### 3.5. Purine-β-Lactam Hybrids

**Figure 6:**
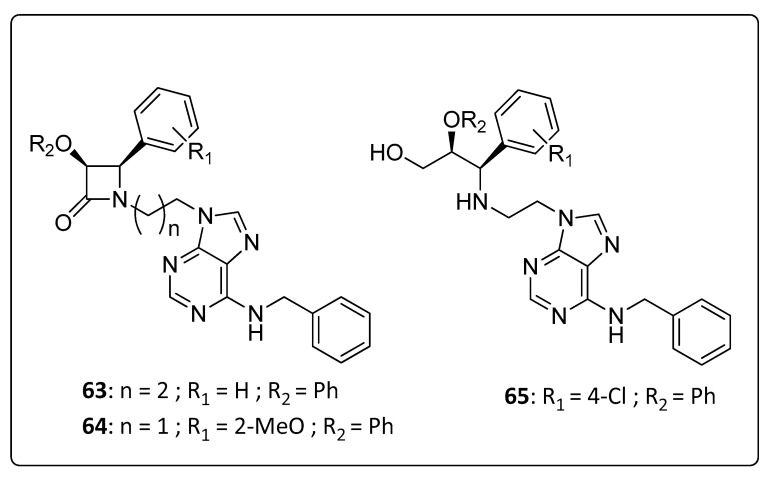
Purine derivatives, **63**–**65**.

The combined synthesis of purines and β-lactams has emerged as a new and relevant strategy in drug discovery programs. Both nitrogen heterocyclic nuclei have been a source of inspiration for medicinal chemists for decades resulting in a variety of derivatives with pronounced biological activities. Purine-based antiviral drugs are largely known, for example, acyclovir, which is used for treatment against herpes [56] and abacavir as an anti-HIV drug [57], whereas β-lactam derivatives are found with anti-tumor activity [58] and anti-malarial activity [59], in addition to being able to inhibit HIV-1 protease [60] and cholesterol absorption [61]. Hence, the objective of D’hooghe and co-authors [62] involved the synthesis and antiviral evaluation of purine-β-lactam hybrids and of the corresponding β-lactam ring-opening products (**63**–**65**; Figure 6). The synthesized hybrids were screened for their antiviral activity against different viruses, including CHIKV. Moderate activities against CHIKV were registered and these initial screenings revealed that purine derivatives, **63** and **64**, were identified as promising lead structures for further study on these scaffolds (Table 5).

### 3.6. Thiazolopyrimidine and Triazolopyrimidine Derivatives

**Figure 7:**
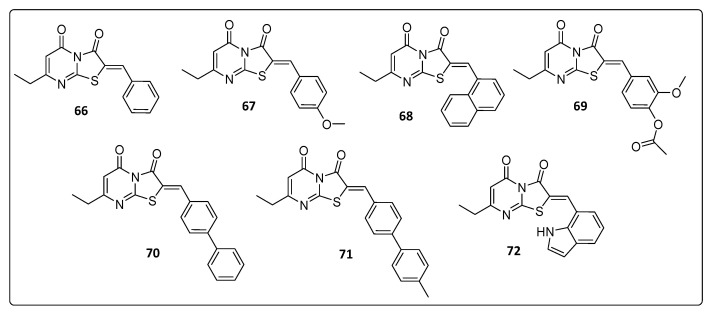
Thiazolopyrimidine derivatives, **66–72**.

In the last years, new nitrogen-based chemical scaffolds have been identified as potential anti-CHIKV agents. Among them, thiazolidinone fused to a pyrimidine ring has been described as a notable pharmacophore for the development of novel anti-CHIKV scaffolds [39]. Figure 7 summarizes the structure of thiazolopyrimidine derivatives with activity against CHIKV. The pyrimidin-3,5(2*H*)-dione (**71**) was the most promising anti-CHIKV compound among the thiazolopyrimidine analogs. This compound was able to inhibit 58% of CHIKV infection at a concentration of 20 µg.mL^−1^, and the EC_50_ was determined using logarithmic interpolation. The calculated EC_50_ of this compound was 42 µM. In addition, three other molecules of this series showed inhibition of CHIKV replication >10% (Table 6) with values of 19%, 28% and 34%, respectively for **66**, **68** and **72** [39].

**Figure 8:**
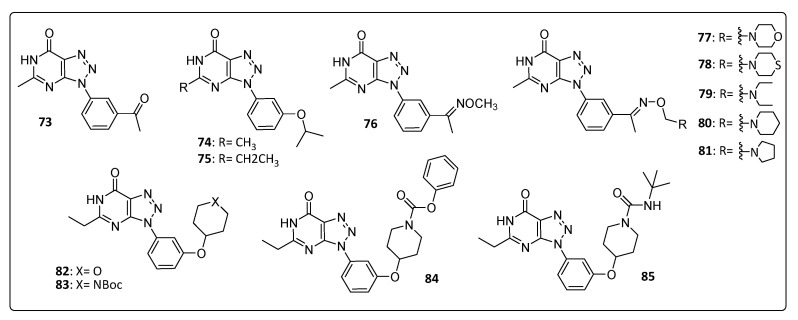
Triazolopyrimidine derivatives **73**–**85**.

Gigante and co-workers [31] synthesized compound **73**, which showed an EC_50_ = 19 ± 2 µM with a CC_50_ > 743 in Vero cells using the CHIKV 899 strain. This triazolopyirimidine derivative **73** and its related compounds **75**, **76**, **77**, **79**, **80** and **81** (Figure 8) were able to inhibit the guanylation step performed by the nsP1 protein of Venezuelan equine encephalitis virus (VEEV), suggesting similar activity against CHIKV. Interestingly, the mutation P34S at nsP1 reverted the inhibition promoted by this compound, conferring resistance against it [11,19].

Compound **75** was ten times more potent than compound **73** and six times more potent than compound **74** in studies of Gigante and collaborators [31]. The introduction of oximes in this nucleus showed significant values of EC_50_ = 5.2 ± 0.5 and 6.9 ± 2 µM for derivatives **78** and **80**, respectively. Notwithstanding this, the best EC_50_ obtained was 2.6 ± 1 µM for molecule **75** only in the CHIKV 899 strain. On the other hand, the compounds **82**–**85** proved to be potent selective inhibitors for CHIKV in all five analyzed strains [19,32]. CHIKV Congo 95 strain was the most susceptible to triazolopyrimidines and exhibited and EC_50_ between 0.3 and 1.0 µM to compounds **82**–**85**. For the St. Martin strain, derivatives **82** and **84** had better efficacy results when compared to compounds **73** and **75** (Table 7) [32].

## 4. Conclusions

The purpose of this review was to provide a significant insight into the development of new nitrogen-based heterocyclic antiviral compounds against CHIKV and their likely mechanisms of action in order to guide our knowledge toward compound optimization. The reviewed molecules could act on several targets, such as enzymes and pathways that are essential for entry, survival, and viral infection; however, interactions with cellular proteins can cause greater cytotoxicity, and this work provided new biological perspectives based on the analysis of the relationship between structure and its activity. It is still necessary to emphasize that the studies were carried out in silico and in vitro, necessitating the future exploration of the potential of application of pyrimidines and their derivatives in vivo followed by clinical trials to develop effective antiviral treatments. Analysis of active antivirals and their beneficial effects in chronic infections of the joints and a possible prophylactic use for travelers and residents of endemic areas will be necessary. The information on molecules presented in this work could be used as the basis for planning new classes of CHIKV inhibitors with expressive selectivity for other pharmacological targets and to identify their mechanisms of action to obtain new antiviral drugs.

## Figures and Tables

**Table 1:** Anti-CHIKV activities of the nitrogen heterocycle–coumarin hybrid in the CHIKV 899 strain.

Reference	Compound	CC_50_ (μM)	EC_50_ (μM)	SI	LogP
Hwu (2015) [34]	7	178	19.1	9.3	1.91
	8	117	10.2	11.5	2.13
	9	30	18.4	1.6	ND
	10	117	54.5	2.2	ND
	11	144	17.2	8.8	−1.07
	12	126	58	2.2	ND
	13	114	26.4	4.3	0.611
	14	107	19.0	5.6	−2.60
	15	60.2	23.1	2.6	0.467
	16	75.2	13.0	5.8	−2.74
	17	104	45.1	2.3	ND
	18	13.8	4.6	3.0	ND
	19	>284	192	>1.5	1.32
Hwu (2019) [54]	20	81.3	40.9	1.98	3.22
	21	>212	9.9	>21.7	3.01
	22	96.5	10.3	9.37	2.97
	23	>227	13.9	>16.3	3.77
	Standard: 3-[(methylthio)-methyl]coumarin	ND	>485	ND	-

EC_50_ is the concentration of a substance that gives half-maximal response (efficacy); CC_50_ is the extract concentration of a substance that reduces the cell viability by 50%; SI is the selectivity index.

**Table 2:** Antiviral activity of the aristeromycin analogs against CHIKV-LS3 [35,36].

Reference	Compound	SAH Hydrolase IC_50_ (μM)	CC_50_ (μM)	EC_50_ (μM)	SI
Yoon (2019) [36]	Standard (1)	1.32	6.3	0.8	7.9
	24	0.37	>100	>100	ND
	25	9.70	1.32	0.53	2.49
	26	1.06	>1.25	0.13	>9.6
	27	4.39	>100	>100	ND
	28	0.76	>100	>100	ND
	29	>100	>100	>100	ND
	30	>100	>100	>100	ND
	31	>100	>100	>100	ND
	32	>100	>100	>100	ND
	33	>100	>100	>100	ND
	34	>100	>12.5	1.95	>6.4
	35	>100	>100	>100	ND
	36	>100	>100	>100	ND
Shin (2020) [35]	37	0.36	>250	0.12	ND
	38	0.37	>100	>100	ND
	39	>100	>100	>100	ND
	40	2.03	>25	>100	ND
	41	3.05	>100	>100	ND

IC_50_ is the concentration of a substance that inhibited 50% of replication.

**Table 3:** Anti-CHIKV activity of the ribonucleoside derivatives.

Reference	Compound	CC_50_ (μM)	EC_50_ (μM)
Slusarczyk (2018) [37]	Standard (5)	>10	0.468
	42	47.02	>200
	43	9.04	>200
	44	>200	>200
	45	81.80	>200
	46	>200	>200
	47	81.29	>200
	48	>200	>200
	49	53.10	>50
Ehteshami (2016) [38]	50	30.6 ^c^ 7.7 ^d^ 2.5 ^e^	0.2 ^a,b^
	Standard (2)	ND	0.3 ^a^ 0.6 ^b^

^a^ = CHIKV infectious model (Asian); ^b^ = CHIKV infectious model East/Central/South African 2006 (ECSA); ^c^ = peripheral blood mononuclear cells; ^d^ = Vero cells; ^e^ = CEM cells.

**Table 4:** Antiviral evaluation of piperazinyl–pyrimidine derivatives against CHIKV in Vero cells [9].

Compound	CC_50_ (µM)	EC_50_ (µM)
**Standard (51)**	122 ± 24	8.7 ± 1
**52**	156 ± 35	14 ± 3
**53**	51 ± 19	5 ± 0.4
**54**	44 ± 19	7.1 ± 0.01
**55**	19 ± 2	5.9 ± 0.2
**56**	95 ± 74	15 ± 2
**57**	181 ± 0.4	10 ± 0.2
**58**	>260	4.0 ± 1
**59**	69 ± 7	2.5 ± 1
**60**	106 ± 69	16 ± 1
**61**	66.4 ± 6	9.5 ± 2
**62**	59 ± 18	3.2 ± 0.2

**Table 5:** Anti-CHIKV activities of purine-β-lactam derivatives, **63**–**65 [62]**.

Compound	CC_50_ (μM)	EC_50_ (μM)	SI
**63**	>98.36	17.11	>5.75
**64**	58.97	13.01	4.53
**65**	71.20	11.51	6.19

**Table 6:** Anti-CHIKV activities of thiazolopyrimidine derivatives, **66**–**72 [39]**.

Compound	% Inhibition at 20 μg/mL	EC_50_ (μΜ)
**66**	19	ND
**67**	4.3	ND
**68**	28	ND
**69**	5.5	ND
**70**	5.0	ND
**71**	58	42
**72**	34	ND

**Table 7:** Triazolopyrimidine compounds with activity for CHIKV strains [19,31,32].

Reference	Compound	SI	CC_50_ (µM)	EC_50_ (µM)					
				CHIKV 899	CHIKV Venturini	CHIKV Congo 95	CHIKV St. Martin	CHIKV OPY	CHIKV nsP1-P34S
Gigante (2014) [31]	73	39 ± 5	>743	19 ± 2	26 ± 2	6.35 ± 0.05	23.9 ± 0.5	28	116 ± 60
	74	ND	>704	12 ± 4	ND	ND	ND	ND	ND
	75	64	>668 [31] 167 ± 94 [32]	3 ± 1	1.43 ± 0.01	0.75 ± 0.35	2.9 ± 0.05	2.6 ± 0.5	>167
Gigante (2017) [19]	76	101 ± 73	3.6 ± 1.3	5.2 ± 0.2	1.1 ± 0.1	4.2 ± 0.8	ND	ND	ND
	77	>729	12 ± 1	6.7	2.5	ND	ND	ND	ND
	78	162 ± 1	5.2 ± 0.5	ND	ND	ND	ND	ND	ND
	79	165 ± 8	16 ± 6	ND	ND	ND	ND	ND	ND
	80	95 ± 40	6.9 ± 2	9.6 ± 1.2	3.0 ± 0.5	14 ± 3.5	ND	ND	ND
	81	166 ± 26	16 ± 1.8	ND	ND	ND	ND	ND	ND
Gómez-Sanjuan (2018) [32]	82	70	84 ± 19	1.2 ± 0.01	1.65 ± 0.5	0.3 ± 0.0	2 ± 0.3	5.2	65 ± 40
	83	18	59 ± 23	3.2 ± 1.0	2.6 ± 1.1	1 ± 0.1	ND	11.5	35 ± 29
	84	>147	>220	1.5 ± 0.3	1.2 ± 0.2	0.35 ± 0.5	1.2 ± 0.5	3.4 ± 1.2	ND
	85	23	201 ± 26	8.9 ± 6.1	2.9 ± 0.4	1.0 ± 0.4	3 ± 2.5	5.4 ± 2.4	23 ± 14
	Standard: Chloroquine	8.1	89 ± 28	11 ± 7	ND	ND	ND	ND	ND
